# Supplementary material for: Non-lithifying microbial ecosystem dissolves peritidal lime sand
Source: Nat Commun. 2021 May 24;12:3037. doi: 10.1038/s41467-021-23006-1 (PMC8144198; doi:10.1038/s41467-021-23006-1)
Supplement: Supplementary file 1 — Supplementary Information [file 41467_2021_23006_MOESM1_ESM.pdf]

Supplementary Information for "Non-lithifying microbial ecosystem dissolves peritidal lime sand," by T. M. Present, M. L. Gomes, E. J. Trower, N. T. Stein, U. F. Lingappa, J. Naviaux, M. Thorpe, M. D. Cantine, W. W. Fischer, A. H. Knoll, and J. P Grotzinger

Supplementary Note 1

Geochemical data from porewater and pond water is provided in the attached spreadsheet, "Supplementary Data 1.xslx." Vibracore localities, descriptions, and core recovery data are in Supplementary Table 1. Literature data for $\delta^{13}C$ values of carbon sources are in Supplementary Table 2. Geochemistry of hypersaline pond waters are similar to many pore fluids but the lack of tidal advection of oxidants and rapid microbial respiration permit evaporation to increase aragonite saturation and support mat calcification (Supplementary Figure 1). Graphic logs of vibracore sedimentology are shown in Supplementary Figure 2 and Supplementary Figure 3. Sediment texture and allochems are indicative of deposition by reworking of shoreface sand to gravel in a restricted interior basin (Supplementary Figure 4). Sediment microtextures include evidence for aragonite dissolution (Supplemental Figure 5). Drone imagery shows evidence of brown, tannic groundwater seeping from the shallow subsurface during ebb tides (Supplementary Figure 6).

Supplementary Tables

Supplementary Table 1: *Coring locations and recovery results*

Core	Date	Latitude (°N)	Longitude (°W)	Barrel length (cm)	Distance to sediment top (cm)	Distance to core top (cm)	Length Recovered (cm)	Compaction (%)	Recovery (%)	Site Description
VC-01	8/3/17	21.28868	71.69667	305	94	120	148	14	80	polygonal mats
VC-02	8/3/17	21.28910	71.69632	305	113	199	106	81	100	blister mats near grainstone berm
VC-03	8/4/17	21.29822	71.70177	305	60	85	223	11	101	polygonal mats at edge of mangroves
VC-04	8/5/17	21.29055	71.70635	305	32	70	240	16	102	muddy algal patch behind tidal bar, in about 30 cm water
VC-05	8/8/17	21.30400	71.68665	305	107	137	168	18	100	blister mats on tidal bar
VC-06	8/8/17	21.29833	71.68725	305	185	205	86	20	86	polygonal mats in middle of tidal bay
VC-07	8/8/17	21.29465	71.68778	200	100	105	93	5	98	polygonal mats in tidal creek behind grainstone berm
VC-08	8/8/17	21.29825	71.68423	305	132	166	138	24	99	smooth mats on small sand lobe in tidal bay

Supplementary Table 2: *Carbon isotope endmember summary*

Endmember	Reported $\delta^{13}C$ values (‰, VPDB)	$\delta^{13}C$ value used as mixing endmember	Reference
Open platform seawater DIC	0.6 – 1.0, avg. 0.85 (n=7)	0.85‰	[1]
Carbonate sand	3.8 – 5.4, avg. 5.0 (n=29)	5‰	this study
Atmospheric CO_2, 1999 monthly global average	-8.023 – -8.012, avg. -8.018 (n=12)	-8‰	[2]
Microbial mat organic matter	-14.3 – -12.3, avg. -13.5 (n=12)	-13‰	[3]
Bulk sediment organic matter	-15.3 – -11.7, avg. -13.7 (n=16)		[4]

Supplementary Figures

Supplementary Figure 1: *Geochemical data from hypersaline pond with encrusted domes at southern end of Little Ambergris Cay.*

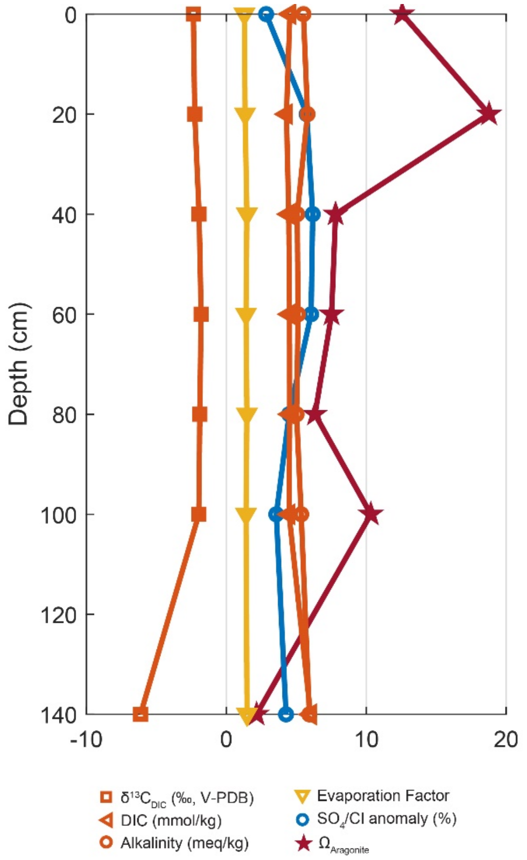

Supplementary Figure 2: *Graphic logs of vibracores VC-01, VC-02, VC-03, and VC-04*

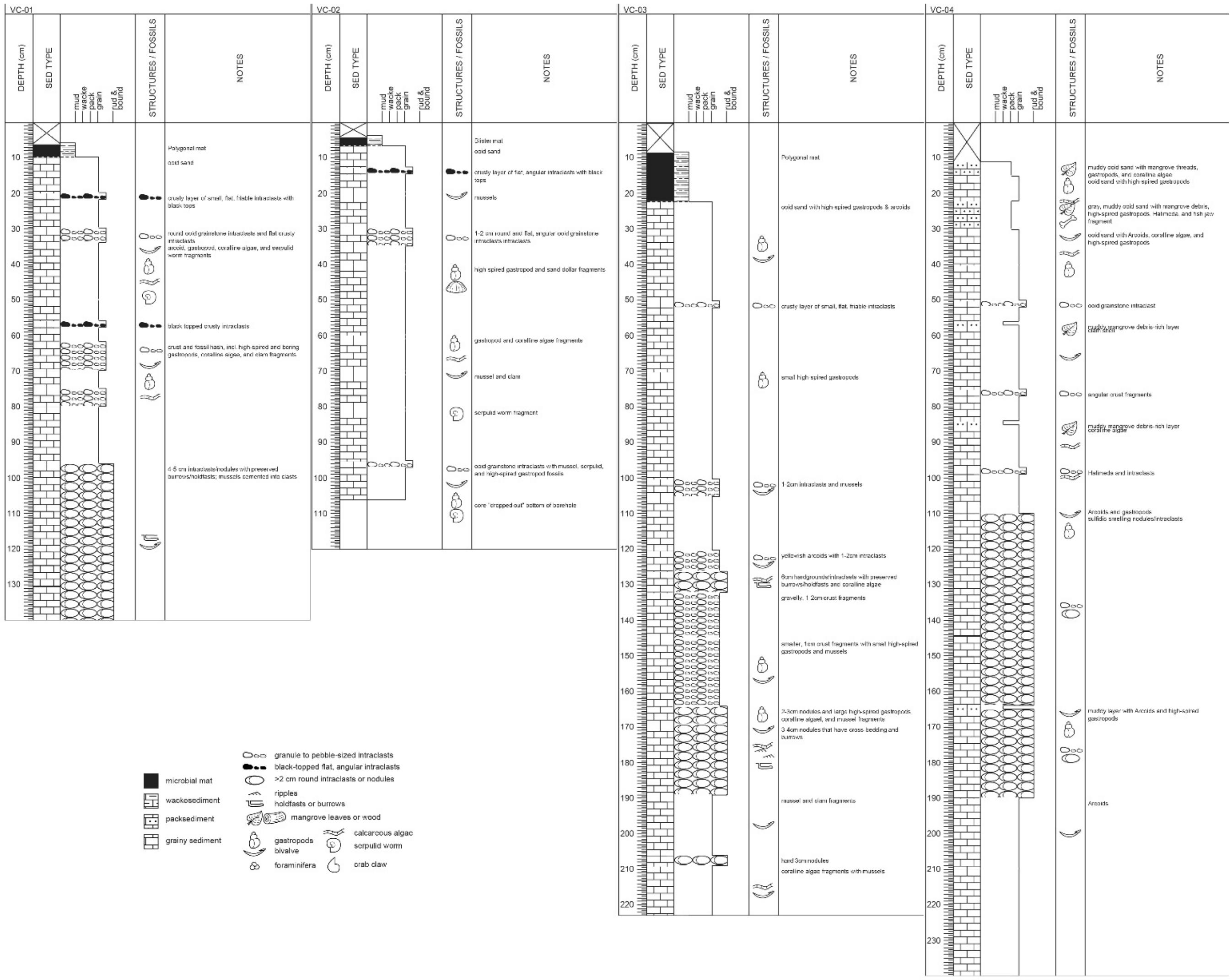

Supplementary Figure 3: *Graphic logs of vibracores VC-05, VC-06, VC-07, and VC-08.*

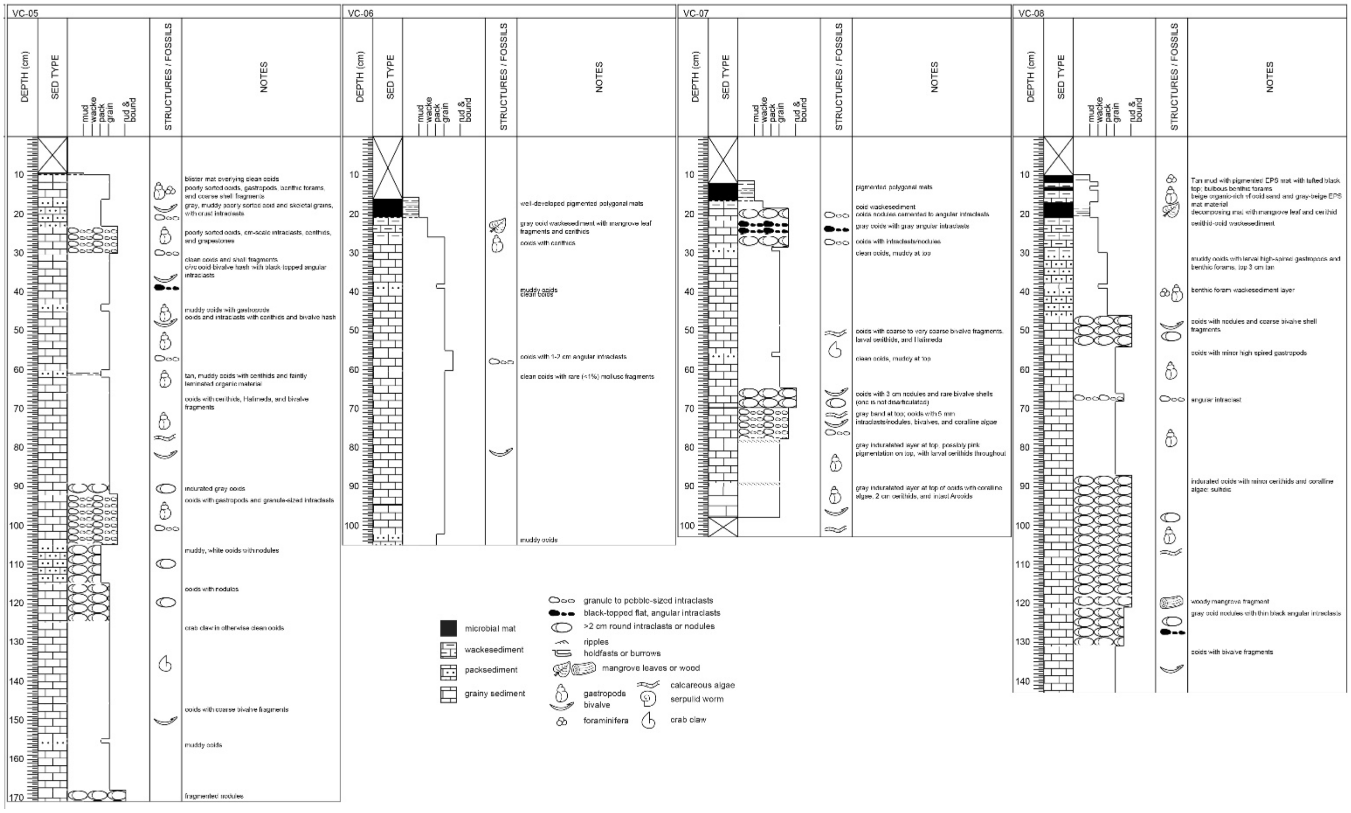

Supplementary Figure 4: *Photos of core sediments. Scales are marked in millimeters.* ***(a)*** *Ooid-bioclast sand, VC-03 at 190 cm.* ***(b)*** *Muddy, ooid-bioclast sand, VC-04 at 165 cm.* ***(c)*** *Black-topped, micro-karsted angular ooid grainstone intraclast interpreted as fragment of crusts forming in interior basin, VC-01 at 20 cm.* ***(d)*** *Rounded ooid-skeletal grainstone intraclast or nodule of partially-indurated sediment, VC-01 at 40 cm.* ***(e)*** *Well-sorted cerithid gastropod-arcoid gravel interpreted as reworked skeletal lag, VC-05 at 35 cm.* ***(f)*** *Large, bleached cerithid gastropod, VC-04 at 160 cm.* ***(g)*** *Olive snail shell, VC-03 at 190 cm.* ***(h)*** *Coralline algae fragments, in skeletal-intraclast gravel, VC-01 at 38-46 cm.* ***(i)*** *Serpulid worm tube with a foraminifera (arrow) in skeletal-intraclast gravel, VC-01 at 38-46 cm.* ***(j)*** *Disarticulated arcoid bivalve in skeletal-intraclast gravel, VC-01 at 38-46 cm.* ***(k)*** *Fragmented bivalve and coralline algae gravel, VC-07 at 80 cm.*

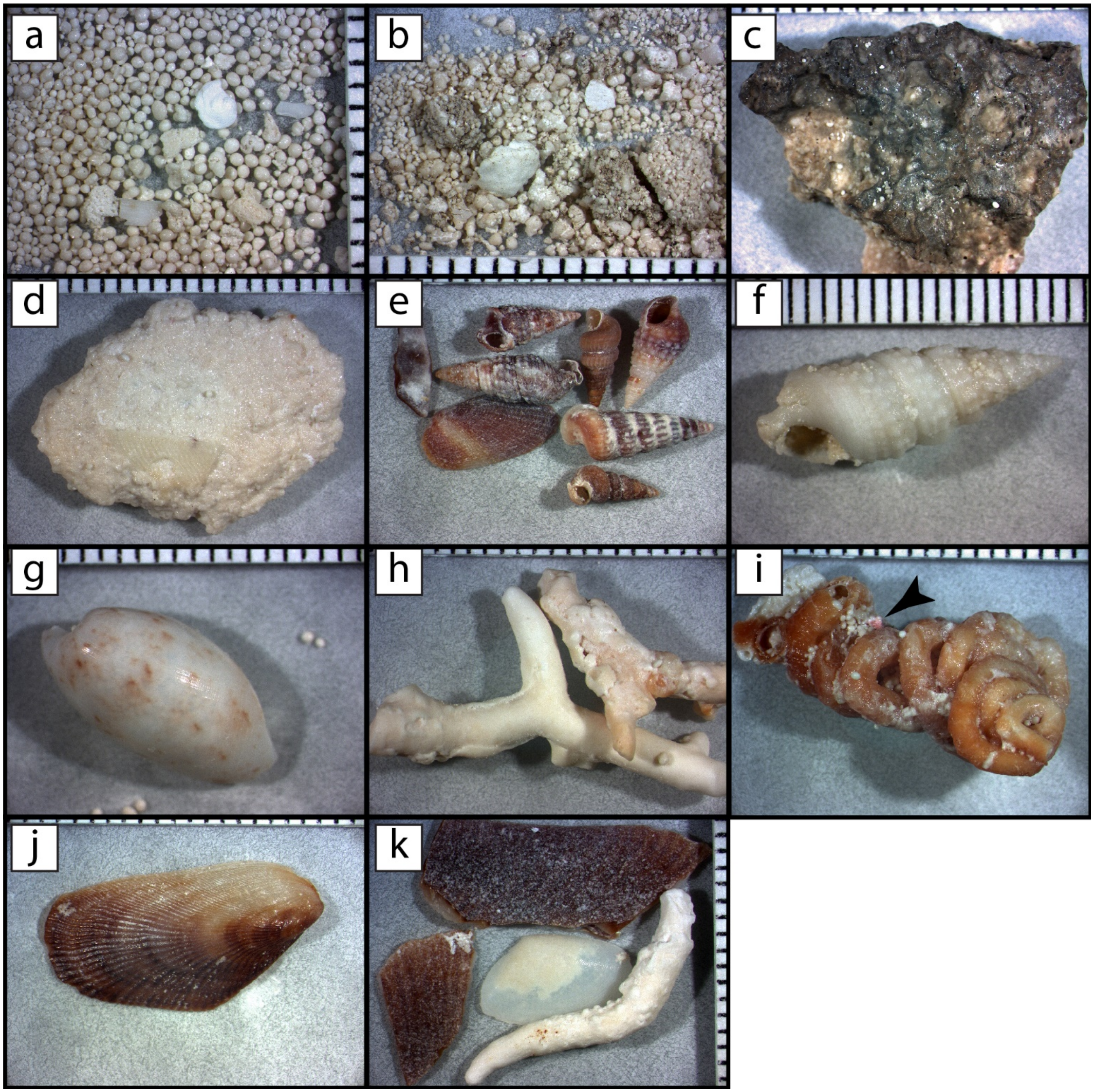

Supplementary Figure 5: *Plane-polarized light photomicrographs of ooid-skeletal sand from VC-03.* ***(a)*** *Solution-enlarged microbores in sand at 60 cm depth.* ***(b)*** *Fabric-selective dissolution of micrite envelope and cement in grapestone grain at 80 cm depth.* ***(c)*** *Solution-enlarged microbores and irregular grain boundaries of grapestone grain at 190 cm depth.* ***(d)*** *Dissolution of algal grain at 200 cm depth.*

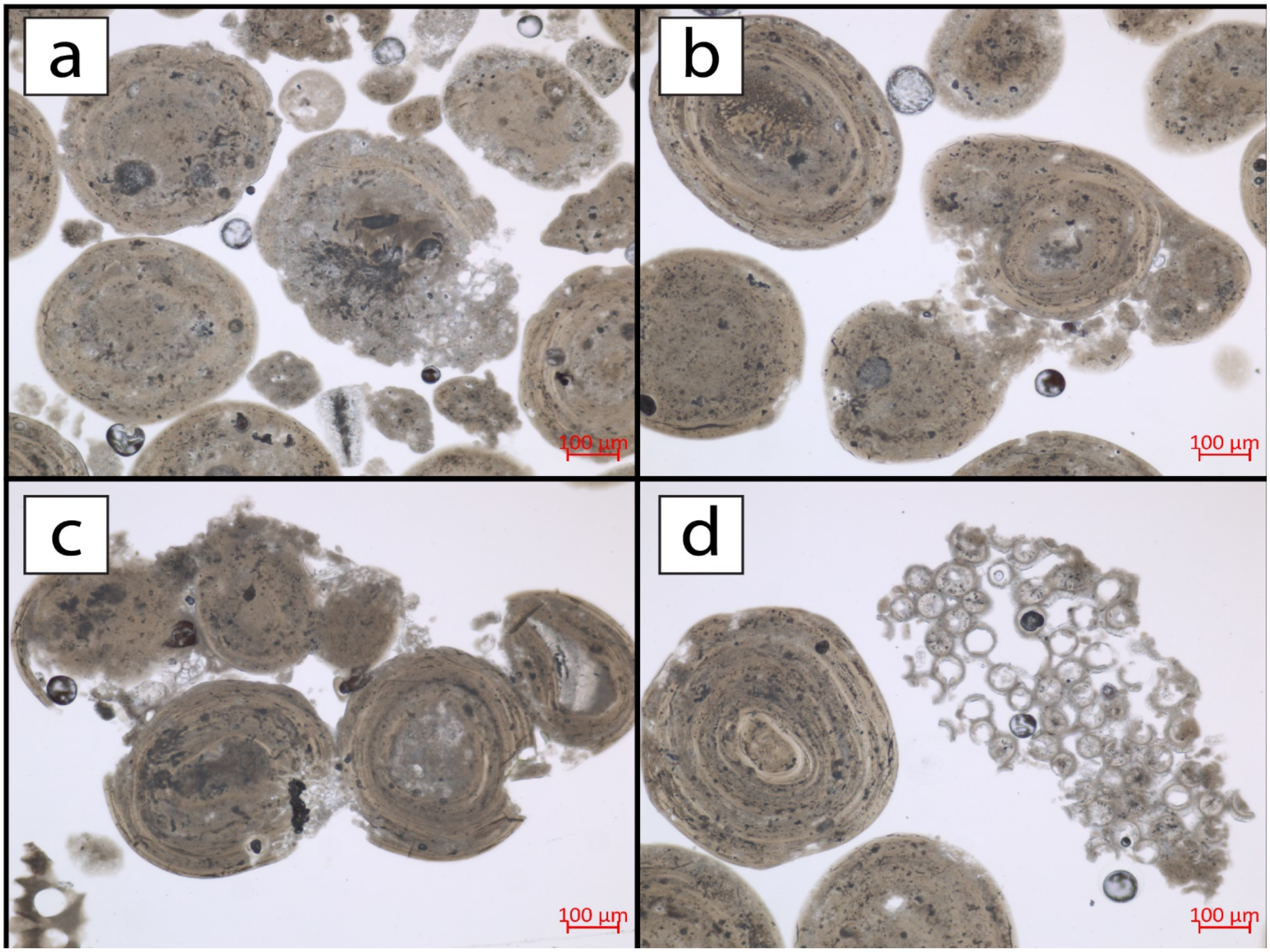

Supplementary Figure 6: *Aerial photograph taken September 24, 2017 of a tidal channel at the western end of Little Ambergris Cay showing brown, tannic groundwater escaping in plumes during ebb tide (arrows). Image acquired with a DJI Phantom 4 drone.*

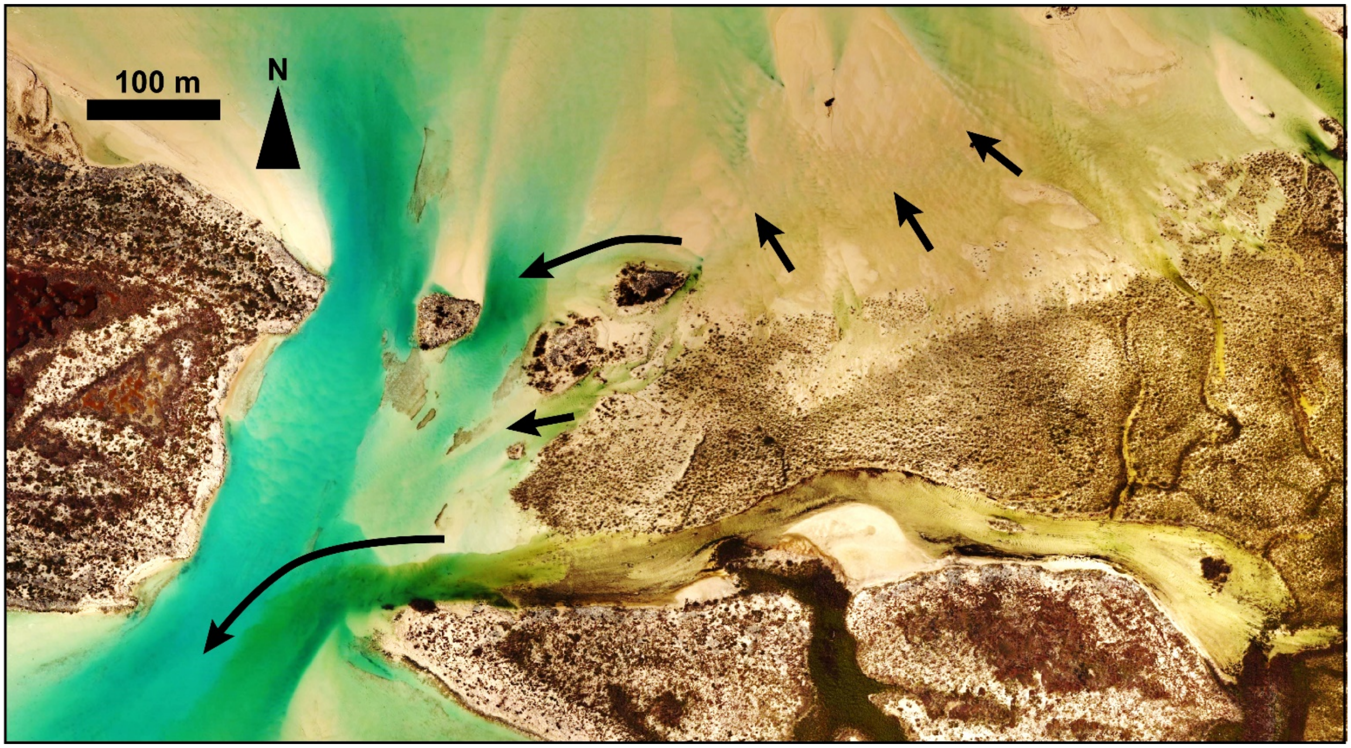

Supplementary References

1. Trower, E. J. *et al.* Active Ooid Growth Driven By Sediment Transport in a High-Energy Shoal, Little Ambergris Cay, Turks and Caicos Islands. *Journal of Sedimentary Research* **88**, 1132–1151 (2018).
2. Keeling, C. D. *et al. Exchanges of atmospheric CO2 and 13CO2 with the terrestrial biosphere and oceans from 1978 to 2000. I. Global aspects*. 88 http://escholarship.org/uc/item/09v319r9 (2001).
3. Gomes, M. L. *et al.* Taphonomy of biosignatures in microbial mats on Little Ambergris Cay, Turks and Caicos Islands. *Front. Earth Sci.* **8**, 1–22 (2020).
4. Raven, M. R., Fike, D. A., Gomes, M. L. & Webb, S. M. Chemical and Isotopic Evidence for Organic Matter Sulfurization in Redox Gradients Around Mangrove Roots. *Front. Earth Sci.* **7**, (2019).
