# Supplementary material for: Non-lithifying microbial ecosystem dissolves peritidal lime sand
Source: Nat Commun. 2021 May 24;12:3037. doi: 10.1038/s41467-021-23006-1 (PMC8144198; doi:10.1038/s41467-021-23006-1)
Supplement: Supplementary file 3 — Description of Additional Supplementary Files [file 41467_2021_23006_MOESM3_ESM.pdf]

Description of Additional Supplementary Files

File Name: Supplementary Data 1.xlsx
Description: Porewater and pond water geochemical data
